# Diagnostic challenges and clinical management gaps in HPV-related oral lesions

**DOI:** 10.3389/froh.2025.1760271

**Published:** 2026-01-12

**Authors:** Gabriela Anaya-Saavedra, Itzel Castillejos-García, Marcela Vázquez-Garduño

**Affiliations:** Oral Pathology and Medicine Postgraduate Program, Health Care Department, Universidad Autónoma Metropolitana, Mexico City, Mexico

**Keywords:** human papillomavirus, koilocytosis, oral cancer, oral lesions, sexually transmitted diseases

## Abstract

The oral mucosa, the gingiva, and the salivary glands are effective reservoirs for HPV. Although HPV-related oral lesions (HPV-OL) have been described since ancient times, their diagnosis and management remain challenging, particularly in people living with HIV. In the oral mucosa, HPV can establish productive or latent infections in basal epithelial cells following microabrasion, resulting in four HPV-OL: squamous papilloma, verruca vulgaris, condyloma acuminatum, and multifocal epithelial hyperplasia, each with characteristic clinical and histological features, though overlapping patterns often complicate diagnosis. While there is strong evidence indicating that HPV can be transmitted through routes other than sexual, misconceptions about sexual transmission and the potential for malignancy continue to persist. Regarding treatment, topical drugs initially designed for the skin or anogenital mucosa lack evidence of safety for the oral mucosa; thus, conservative surgical excision remains the main option. HPV vaccination may contribute to reducing both low- and high-risk HPV infections, with potential impact on related diseases.

## Introduction

The oral mucosa, the ductal epithelium of salivary glands, and the gingival pockets, especially when they are inflamed, are effective reservoirs for human papillomavirus (HPV) (1); while in the head and neck region, the oropharynx represents the preferred site (2). Papillomavirus has been associated with the development of hyperplastic lesions in skin and mucosa (1, 3), and is the etiological agent of malignancies in both the anogenital tract (2, 4) and the oropharynx (2).

The research on HPV has significantly advanced since the last century, with the identification of the causal relationship between HPV and the development of cervico-uterine, anogenital, and ultimately, oropharyngeal cancers (5). Currently, over 400 HPV types have been identified, of which 200 are recognized by the International Committee on Taxonomy of Viruses (4).

HPV-related oral lesions (HPV-OL), also known as oral warts, have been described since ancient times (6, 7); however, diagnosing and managing these lesions remain challenging (8–10), especially among people living with HIV (PLWH), in whom the lesions may show a clinicopathological overlap, as well as a heterogeneous molecular profile (11–13). A key concern is the lack of evidence-based management protocols, which leads to uncertainty in this area of oral medicine.

### Human papillomavirus infection

Papillomaviruses are small, non-enveloped viruses present in both animals and humans. They contain a double-stranded circular DNA that is approximately eight kilobases long, with the capacity to establish: (1) Productive infections, characterized by the expression of viral genes and the assembly of virion particles in differentiated keratinocytes, or (2) Latent infections, in which the viral genomes remain as episomes within the basal or parabasal keratinocytes, exhibiting low replication (5, 10, 14).

**Figure 1:**
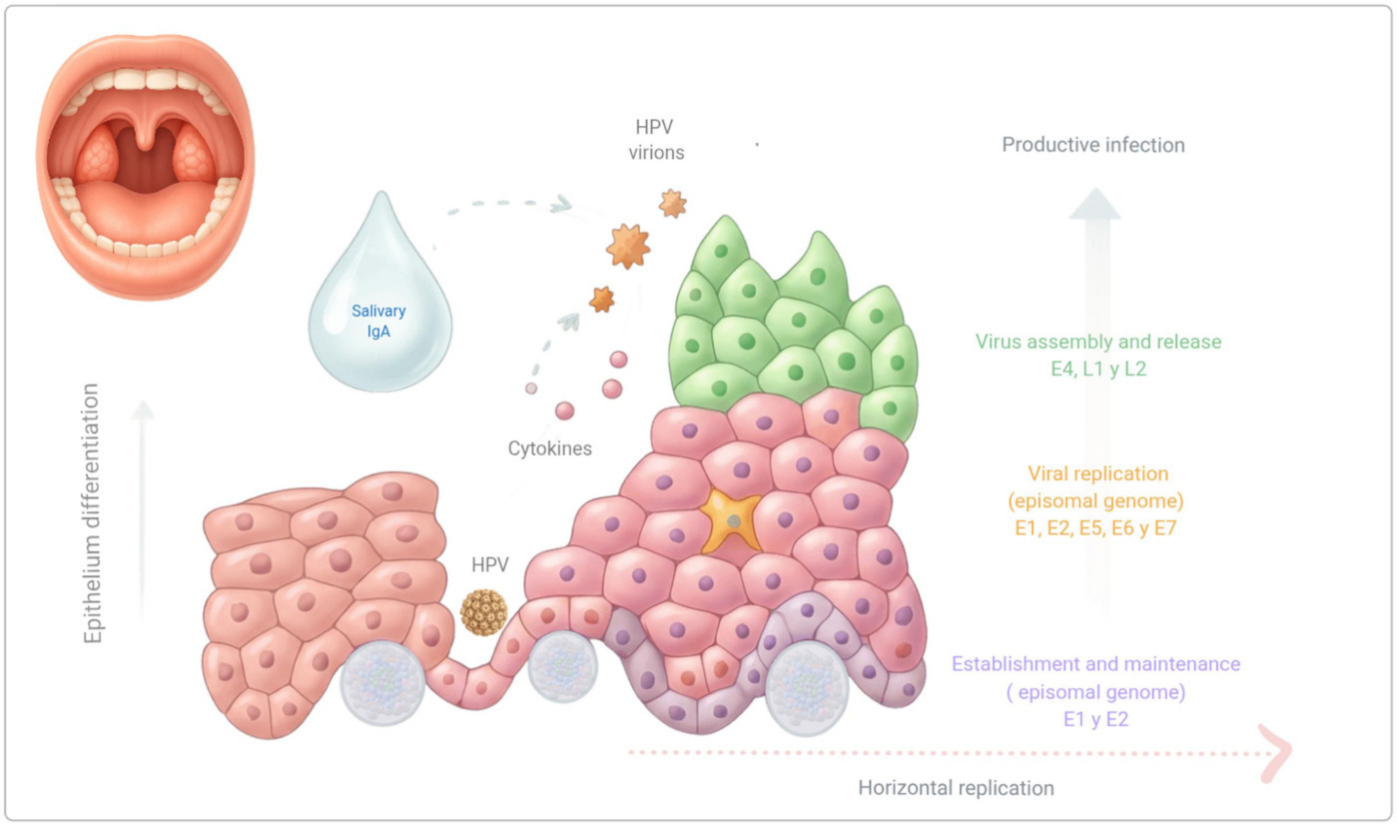
The viral cycle in HPV-related oral lesions. Once the infection establishes in the basal cells, replication depends on epithelial differentiation, with the infection sustained by the normal proliferation of basal cells (horizontal replication). Viral genome amplification occurs suprabasally in red keratinocytes (vertical replication), while the virus assembles in the outermost layers. The immune microenvironment of the oral mucosa, including cytokines, IgA, and mucosal-associated lymphoid tissue, facilitates a productive viral cycle without triggering a strong inflammatory response.

Since both the mucosa and the epidermis function as mechanical barriers, HPV particles can infect only basal epithelial cells through epithelial wounding or microabrasions (4, 10). Once inside a host cell, the episomal HPV genome rapidly replicates, producing between 10 and 200 copies/cell, initiating the establishment phase (4), and later proliferates within the epithelium using the two types of basal cell division: a symmetric growth that allows horizontal replication by basal epithelial cells proliferation, and an asymmetric growth that produce daughter cells that move toward the upper epithelial layers in productive infections (15, 16) (Figure 1).

The HPV-DNA genome comprises three regions: an early region that encodes the early proteins E1, E2, E4, E5, E6, and E7; a late region that encodes the viral capsid proteins L1 and L2; and the long control region that regulates viral replication and transcriptional and post-transcriptional activity (4, 5, 15, 17). Based on genomic variations in the L1 open reading frame, HPVs are classified into five genera: *Alphapapillomavirus, Betapapillomavirus, Gammapapillomavirus, Mupapillomavirus,* and *Nupapillomavirus*, with most types belonging to the first three genera (ɑ, *β*, ɣ) (2).

HPV genotypes are also classified by their behavior and oncogenic potential into low-risk HPV (LR-HPV), which may persist latently as reservoirs on basal cells, and high-risk HPV (HR-HPV), which can integrate its DNA into the host genome (17–20). LR-HPV genotypes are associated with benign skin and mucosal lesions, including squamous papilloma, sinonasal exophytic papillomas, verruca vulgaris, multifocal epithelial hyperplasia, and laryngeal papillomatosis. In contrast, HR-HPV, particularly types 16 and 18, is associated with mucosal malignancies at both anogenital and head-and-neck sites (2, 4, 21).

HPV infection can express in three forms: clinically, subclinically, and latently. In the latter two forms, individuals do not exhibit clinical lesions; the interval between initial HPV exposure and symptom onset ranges from 3 weeks to 8 months. In approximately 30% of cases, lesions may spontaneously regress within a few months, an event associated with increased activity of cutaneous CD4 + lymphocytes (15).

Also, some HPV genomes may be lost following the onset of cytopathic changes, a phenomenon referred to as the “hit-and-run” theory, which could explain the variability observed in HPV detection assays. In this scenario, a transient HPV infection (“hit”) can induce changes that persist through cell divisions, even after the virus has been cleared (“run”) from the body (22).

### The four types of HPV-OL

In the oral mucosa, the epithelial growths caused by HPV can resemble other reactive, inflammatory, and hyperplastic conditions (23–26); therefore, a clinical diagnosis of “HPV-related oral lesion” should be considered provisional and must be followed by a histopathological examination to confirm the presence of cytopathic changes and to rule out any dysplastic or reactive modifications (24, 27).

The four HPV-OL are squamous papilloma (SP), verruca vulgaris (VV), multifocal epithelial hyperplasia (MEH), and condyloma acuminatum (CA), each with a unique clinicopathological profile (3, 8, 27). Although HPV-OL arise from the same pathogenic process, individual factors such as age, anatomic site, local trauma, and the host immune response can influence both clinical and histological patterns (8, 10); thus, in some cases, achieving an accurate diagnosis may be challenging (24, 28).

SP is the most common type of HPV-OL, and typically appears as a solitary, pedunculated growth that exhibits “finger-like” or “cauliflower-like” projections, ranging in color from white to pink, and rarely exceeding five millimeters in size. The most affected oral SP sites are the soft palate and the tongue (8, 24, 27, 29). Histologically, the lesion presents papillary fronds of vascular connective tissue covered by a stratified squamous epithelium, with a sparse population of koilocytic cells; the mitotic activity is confined to the basal and parabasal layers, and dysplastic or atypical features are absent (3, 8, 24).

VV primarily occurs in children and adolescents, and is often associated with autoinoculation from other body sites (27, 29). Potential VV triggers include inflammation or trauma; thus, some authors support classifying the lesion as reactive rather than infectious (24). Clinically, it appears as a small, sessile, well-defined papillary nodule, ranging in color from pink to white, typically affecting the labial mucosa, anterior tongue, and palate (24, 27, 29, 30). Histologically, VV is similar to cutaneous warts, showing exophytic hyperkeratotic projections with inward cupping of the rete ridges, which creates a “church-spire” keratin pattern. VV also exhibits a well-developed granular cell layer, along with mitosoid figures and koilocytes in the superficial epithelial layers (3, 24, 27). In some cases, the verrucous architecture may clinically resemble that of verrucous carcinoma, highlighting the importance of biopsy and histopathological analysis (24).

CA, is a rare form of HPV-OL that primarily affects adults; it is typically larger and more numerous than papillomas, often appearing on the tongue, lips, and palate (3). This sessile lesion exhibits a broad base and a cauliflower- or moruloid-surface appearance; in some cases, multiple lesions coalesce into larger plaques (8, 30). Histologically, CA is characterized by a papillary epithelium with markedly acanthotic cells and bulbous, broadened rete ridges. Parakeratin often fills deep clefts between papillae, and koilocytes are prominent in the upper layers (10, 31, 32). Although condylomas may superficially resemble papillomas or verrucae, they can be distinguished by their larger size, tendency to present in groups, and a sessile base, which serve as key diagnostic features (3, 27).

MEH, formerly known as Heck´s disease and also recognized as focal epithelial hyperplasia (33). Although this condition has been observed in adults, the lesions primarily occur in individuals aged 5–15, with females more frequently affected. MEH has been reported in specific populations, including indigenous groups in South America, Native Americans, Mexican Indigenous people, Eskimos, and Sub-Saharan African children (10, 29, 34–38), some of them showing a high prevalence of HLA-DR4 alleles, a genetic susceptibility that has been studied in small cohorts (39). Clinically, MEH presents as well-defined round or oval papules of the same color as the surrounding mucosa that range in size from 0.1 to 0.5 cm in diameter and often merge into larger nodules. Although lesions can appear anywhere on the oral mucosa, the most commonly affected sites are the lips, buccal mucosa, tongue, and commissures (33, 35). A notable characteristic is that nodules tend to disappear when the mucosa is stretched, but reappear once the tension is released (33, 36, 38, 40). Histopathologically, MEH is characterized by basal hyperplasia with interconnecting rete ridges, along with variable numbers of koilocytes and mitotic figures (27, 35, 36). Unlike other hyperplastic and reactive oral mucosal lesions, most MEH cases resolve completely without treatment (37); however, some chronically inflamed lesions can lose their architecture and mimic conditions such as CA or SP (41, 42). Moreover, in PLWH, MEH may also exhibit abnormal keratinization and harbor multiple HPV genotypes, including high-risk types (12, 41, 43).

### The diagnostic challenge

As mentioned, the clinicopathological profile of HPV-OL may vary, and the criteria may be unclear and subject to change (8). Despite efforts to classify each HPV-OL appropriately, it is noteworthy that this academic classification (SP, VV, MEH, and CA) rarely influences treatment decisions, which in most cases involve surgical excision (44, 45).

In microscopical terms, although koilocytosis is recognized as a sign of cytopathic viral damage, it is not consistently present (9, 46, 47). The presence of koilocytes may be influenced by the HPV viral load, the stage of the viral cycle, and the specific oncogenic potential of the HPV type, as HR types rarely induce koilocytic changes (46, 48).

Regarding genotypification, despite each HPV-OL has been linked to specific types (SP: HPV-6,-11; VV: HPV-2,-4; CA: HPV-6,-11,-16,-18; MEH: HPV-13,-32) (1, 3, 8, 29), a number of studies have reported the presence of multiple HPV infections, including HR genotypes (3, 29, 43, 49, 50). HPV typing can be valuable for studying the virus's distribution and understanding its pathogenic mechanisms; additionally, knowing the specific HPV type may be beneficial for immunosuppressed patients with atypical HPV-OL. However, even in these situations, genotyping serves as supplementary information and should not be used as a diagnostic tool (51).

### The myth of the sexual transmission

A common misconception among clinicians and patients is that HPV-OL could imply sexual transmission (52, 53). According to scientific evidence, since the presence of an HPV infection in the oral mucosa could imply sexual transmission or represent a concurrent genital infection, most HPV oral infections include horizontal transmission (autoinoculation and heteroinoculation), and potentially indirect transmission through contaminated objects (fomites) like towels, clothing, toilet seats, and medical devices (3, 29, 52–54); additionally, perinatal transmission a reliable explanation for HPV oral presence in newborns (10, 53, 54).

Studies in mice have indicated that HPV can persist on surfaces and remain infectious for up to 8 weeks, and it can also survive in exfoliated epithelial cells for up to 1 year (55). Research has demonstrated that HPV is resilient to drying, maintaining 30% of its infectivity even after 7 days of dehydration. However, when HPV particles are transferred from one surface to another, viral concentration decreases significantly (10-fold), thereby reducing infectivity (55). Disinfectants that contain ethyl or isopropyl alcohol solutions, as well as octenidine, are ineffective against HPV; in contrast, the virus is susceptible to hydrogen peroxide, ortho-phthalaldehyde, and UV-C light (5).

### The myth of malignant transformation

Current evidence does not support a malignant potential for HPV-OL (8, 10). As revised, most HPV-OL are associated with low-risk HPV types (8, 10, 43, 47), which are typically found in an episomal state, allowing normal epithelial maturation without the genomic instability that led to dysplastic changes or malignant transformation (10, 29, 47, 56). The thorough understanding of HPV in the anogenital area has shown that HPV infection is necessary but not sufficient for the development of cancer (16, 57). According to Demarco et al. (2020) (58), approximately 80% of HPV cervico-uterine infections are cleared by the immune system within 3 years, and only 3% progress to malignancy within 7 years. Misinformation regarding HPV-OL may generate anxiety and lead to aggressive treatments; thus, clear communication about the benign nature of oral HPV is essential to promote a conservative therapeutic approach. Also, it is crucial to distinguish oral lesions caused by HPV from the so-called HPV-associated epithelial dysplasia, a separate histopathological entity with malignant potential (23, 59).

### Oral HPV lesions in people living with HIV

People living with HIV have an increased risk of oral and oropharyngeal HPV infection and of developing HPV-OL (60–63). It has been suggested that several factors, including aging, the use and duration of antiretroviral treatment, an impaired immune response despite ongoing antiretroviral therapy, and the interaction between HIV-HPV, may influence this increased risk (10, 12, 64, 65). Earlier studies have reported the increased risk of HPV + oropharyngeal tumors in PLWH compared to people without HIV (66, 67), with poorer survival rates and worse outcomes (68). Thus, close monitoring is necessary, particularly in those with additional oral cancer risk factors, such as tobacco and/or alcohol consumption.

### Therapeutic perspectives of HPV-related oral lesions

As shown in Table 1, most studies on HPV-OL management are anecdotal, presenting case reports or small case series. The therapy approaches vary significantly, demonstrating that therapeutic decisions are influenced by local practices and clinical consensus than by evidence-based medicine (37, 40, 69).

**Table 1:** Overview of treatment options for HPV-related oral lesions with assigned levels of evidence (Oxford CEBM, 2009).

Treatment	Description	Level of evidence	Study type	References
Observation	Periodic monitoring without immediate intervention Based on clinical opinion/experience	V IV	Case report (*n* = 1) Case series (*n* = 6)	Mansouri et al. (40) Nartey et al. (37)
Surgical excision	Removal of oral lesions through a scalpel Surgical excision with an Er: YAG laser	V V	Case report (*n* = 1) Case report (*n* = 1)	Sarfi & BenYahya (44) Wang et al. (45)
Cryotherapy	Application of liquid nitrogen to freeze and remove lesions	IV	Case series (*n* = 9)	Ledesma-Montes et al. (33)
Laser diode surgery	Therapeutic approach for tissue ablation using special light waves	V V V	Case report (*n* = 1) Case report (*n* = 1) Case report (*n* = 1)	Goswami et al. (71) Misir et al. (72) Sarabadani et al. (42)
Electrocauterization	Surgical approach when an electric current is applied to burn a lesion	V	Case report (*n* = 1)	Al-Zahawi et al. (70)
Photodynamic therapy	Administration of a photosensitizing agent (5-aminolevulinic acid) followed by specific wavelength light to induce selective damage to infected cells.	V V	Case report (*n* = 1) Case report (*n* = 1) Case series (*n* = 2)	He et al. (92) Tian et al. (91) Li et al. (93)
Pharmaceutical agents	Trichloroacetic acid Interferon-alpha Interferon-beta Topical cydofovir	IV IV V V V V	Prospective cohort (*n* = 20) Case series (*n* = 4) Case report (*n* = 1) Case report (*n* = 1) Case report (*n* = 1) Case report (*n* = 1)	Carmona-Lorduy et al (86) Lozana-Nur et al. (84) Akyol et al. (83) Steinhoff et al. (85) Beaulieu et al. (80) DeRossi & Laudenbach (82)
	Imiquimod: immunomodulator and Toll-like receptor 7 agonist Intralesional immunotherapy (measles, mumps, and rubella vaccine)	V IV IV V IV V	Case report (*n* = 1) Case series (*n* = 3) Case series (*n* = 2) Case report (*n* = 1) Case series (*n* = 3) Case report (*n* = 1)	Méndez-Flores et al. (75) Gemigniani et al. (76) Esquivel-Pedraza et al. (77) Barikbin et al. (78) Yasar et al. (79) Santana-Gutiérrez et al. (81)

Surgical conservative excision, followed by histopathological analysis to confirm the diagnosis, remains the main treatment option (44, 45). Other studies recommended eliminating of the lesions using physical methods such as cryotherapy, electrocauterization, CO₂ laser, and diode laser (33, 42, 70–72), methods that can destroy tissue and compromise the histopathological evaluation.

In the case of MEH, as the condition is self-limiting and tends to resolve with age, observation is the better approach to manage it. If MEH does not cause functional or cosmetic issues, no treatment is necessary (33, 73); however, if the lesions impede normal biting and chewing, are painful, or raise aesthetic concerns or social stigma (29), surgical excision may be warranted.

Another concern is the oral use of topical drugs designed for the skin or anogenital area, such as imiquimod, trichloroacetic acid, retinoic acid, interferon, isotretinoin, glycyrrhizin acid, immunotherapy, and cidofovir (74–86). Oral mucosa differs significantly from anogenital mucosa and skin; thus, it is essential to emphasize that these anatomical areas have distinct biological environments, are exposed to different stimuli, exhibit dissimilar permeability and microbiota, and exhibit distinct healing dynamics (87). Despite the lack of evidence supporting its safe use on the oral mucosa, several case reports reported successful responses with 5% imiquimod for HPV-OL (and other oral diseases) (88–90). However, there are no established recommendations for the optimal imiquimod dosage for HPV-OL; moreover, manufacturers do not recommend its intraoral application due to the potential for systemic absorption and the risk of ingestion. Unfortunately, oral ulcerations following the oral use of imiquimod have been reported (74, 75).

On the other hand, photodynamic therapy has emerged as a potential alternative to HPV-OL, particularly in extensive lesions, recurrent condylomas, or lesions located in anatomically complex areas, such as the soft palate (91). It is essential to highlight that, to date, evidence of its efficacy and safety is limited, coming from small case series with short follow-up periods and without standardized dosing parameters (91–93).

Other emerging options for treating refractory cutaneous and genital warts include intralesional immunotherapy targeting Candida sp. antigens and the MMR vaccine, which stimulates Toll-like receptors and enhances cytokine production, particularly interferons. The results of these case series are heterogeneous, with HPV-OL resolution rates of 26%–92% for MMR treatment, 39%–88% for Candida antigens, 23%–94% for PPD, and 33%–70% for BCG (5, 81).

To date, three HPV vaccines have been approved by the Food and Drug Administration and the European Medicines Agency: Cervarix (against HPV-16 and −18), Gardasil (against HPV-6, -11, -16, and -18, and the nonavalent Gardasil 9 (against HPV-6, -11, -16, -18, -31, 3-3, -45, -52, -58 (94).

Many countries have established HPV vaccination campaigns to prevent the infection and HPV-related diseases (5). Initially, the vaccines were recommended to girls, but recently, the campaigns have expanded to include a diversity of genders and groups of ages (95); however, several factors, such as vaccine coverage, can influence the vaccination's effectiveness in reducing HPV rates, targeting specific HPV types, and determining vaccine efficacy (96).

In relation to the head and neck region, increased vaccine coverage is expected to impact the rates of oropharyngeal cancer and some HPV-OL (29, 97); a recent study suggests that the nonavalent vaccine may provide protection for oral cancer diagnosis, and the quadrivalent vaccine may be sufficient to prevent oral potentially malignant disorders and HPV-OL (97).

In summary, based on current evidence and its gaps, several principles can be stated:

- Both clinical and histopathological characteristics must guide the diagnosis
- Conservative surgical excision is the better treatment option so far.
- Most MEH requires no treatment in immunocompetent patients.
- Genotypification is not necessary for HPV-OL diagnosis.
- Topical treatment should never be first-line therapy.
- HPV-OL recurrences in PLWH are expected; surveillance is essential.
- There is an urgent need for validated clinical trials on HPV-OL treatment.
- HPV vaccination can potentially reduce HR and LR-HPV infections, thereby impacting HPV related diseases.
